# Revisiting the wrinkling of elastic bilayers I: linear analysis

**DOI:** 10.1098/rsta.2018.0076

**Published:** 2019-03-13

**Authors:** Hamza Alawiye, Ellen Kuhl, Alain Goriely

**Affiliations:** 1 Mathematical Institute, University of Oxford, Oxford, UK; 2 Living Matter Laboratory, Stanford University, Stanford, CA, USA

**Keywords:** growth, compression, instability, nonlinear elasticity, buckling, wrinkling

## Abstract

Wrinkling is a universal instability occurring in a wide variety of engineering and biological materials. It has been studied extensively for many different systems but a full description is still lacking. Here, we provide a systematic analysis of the wrinkling of a thin hyperelastic film over a substrate in plane strain using stream functions. For comparison, we assume that wrinkling is generated either by the isotropic growth of the film or by the lateral compression of the entire system. We perform an exhaustive linear analysis of the wrinkling problem for all stiffness ratios and under a variety of additional boundary and material effects. Namely, we consider the effect of added pressure, surface tension, an upper substrate and fibres. We obtain analytical estimates of the instability in the two asymptotic regimes of long and short wavelengths.

This article is part of the theme issue ‘Rivlin's legacy in continuum mechanics and applied mathematics’.

## 1. Introduction

In the development of rational mechanics after WWII, there was much emphasis on foundations, abstraction and formalism. Yet, by its very nature and applicability to the world around us, mechanics is not an abstract concept. In this era, Ronald S Rivlin stood out as a pragmatic researcher, as he was interested in identifying new phenomena, in conducting experiments and in developing general methods to tackle scientific problems. He was at heart a problem-solver and his enduring legacy is to have given us the tools to understand the world around us. Two of his contributions are particularly noteworthy in this regard. First, he systematically derived exact solutions for simple geometries (cuboids, spheres, cylinders [1]), and second, together with Green, Shield and Pipkin [2,3], he developed a general method, *the small-on-large theory*, to obtain both vibration modes and stability regimes for mechanical systems in large deformations.

The present contribution follows in Rivlin's footsteps as it uses both ideas to their full potential by presenting a general theory for the stability of a bilayer system in large deformations.

### (a) Multi-layered elastic materials

Multi-layered elastic materials have been widely studied in engineering literature with applications ranging from construction materials [4] to three-dimensional printing. The past few decades have seen a resurgence of interest in the development of a mathematical description of these structures, moving from the classical linear theory of elasticity to a more accurate nonlinear hyperelastic description that is valid for the large deformations relevant in many biological contexts. Additionally, where traditional analyses often made use of thin-film approximations [5] or extreme ratios of stiffness between layers, newer works have begun to explore the intermediate parameter space.

Owing to the complex nonlinearities present in this problem, it is often too difficult to compute the solution of boundary-value problems explicitly in this setting. As such, we must often make use of indirect methods such as perturbation expansions to identify bifurcation points in the system as parameter values are changed, as well as the stability of any non-trivial solutions that may emerge. Such methods have been used to great success to compute the critical uniaxial compression required to cause buckling of an elastic half-space coated in a thin, stiffer elastic film [6]. Following works have considered variations of the physical setting such as pre-stretching the substrate [7], further compressing the buckled bilayer to induce a second, periodic-doubling bifurcation [8], the limiting behaviour of the system as the stiffness ratio of the layers tends to unity [9], the effect of adding reinforced fibres to the substrate [10] and the replacement of compression with growth as a mechanism to induce buckling [11].

This final modification is of particular relevance in the study of biological materials, as it allows us to model the formation of complex organs such as the brain *in utero* [12]. The physical structure of mammalian brains consists of distinct layers of cells with similar, but different mechanical properties and thicknesses. In particular, we can divide the brain into the outer layer of grey matter (the cortex), which primarily consists of neuron cell bodies, and the inner white matter (the subcortex), which primarily consists of axons and their insulating myelin sheaths. The process that leads to the evolution of the characteristic convolutions (gyri) of the brain has only recently seen convincing explanations after several decades of scientific research [13]. There are several competing mechanical theories [14] that seek to describe the evolution of cortical folds, but all current evidence points towards differences in the growth rates in the layers as the driving force behind cortical folding [11,15–17]. In particular, it was recently demonstrated that the wrinkling instability also correctly captures variations of thickness between gyri and sulci [18].

Experimental verification of these theories is currently limited due to the difficulties involved in acquiring and mechanically testing brain matter *in utero*, but advances in non-invasive imaging techniques may provide the data needed to better validate their predictions [19,20]. Recently, it has been demonstrated that it is possible to capture the mechanical response of brain tissue in an elasticity-based framework [21]. Furthermore, preliminary numerical simulations have been able to demonstrate—at least phenomenologically—brain morphogenesis in this framework (see [22,23]).

### (b) Mathematical studies of compression-induced instabilities

One of the first mathematical studies of wrinkling in elastic solids came from Biot in his seminal paper of 1963 [24]. In this work, he considers an incompressible elastic half plane under uniform compression of two different types, each of which induces a *surface instability*—at some critical value of compression, a family of deformations that display periodic oscillation localized to the surface of the material become viable solutions of the equilibrium equations of elasticity. The wavelength of the oscillations along the surface of the material is undetermined by the theory as there is no possible choice of a length scale for an infinite half plane.

A slightly different approach to wrinkling and similar pattern formation phenomena comes from works of plates bounded on a substrate [25,26]. In these works, elastic sheets bonded to elastic substrates are modelled using a variational form of the von Kármán plate equations (which can be derived from full three-dimensional nonlinear elasticity [27]) and wrinkling is identified as the result of competition between minimization of the non-convex membrane energy and the regularizing bending energy. In particular, scaling laws of the energy with respect to thickness of the elastic sheet were identified and it was demonstrated that this fitted with characteristic wrinkling patterns seen in the physical world [28,29]. While these studies provide precise estimates, they have a limited (and well acknowledged) range of validity regarding properties of the displacement field of the plate which cannot capture some of the phenomenology we see in thick, multi-layered elastic media.

Another important related phenomenon in the theory of soft solids is *creasing*, where a sharp, self-contacting region forms almost instantaneously when a critical compression is exceeded. Experimentally, this is seen to occur at a lower critical strain than that predicted by Biot's analysis [30] and, in recent years, an understanding of this phenomenology as a separate elastic surface instability has been developed [31]. This has come from both numerical studies (see [32,33]) and recent asymptotic analyses [34,35], which address the mathematical difficulties involved in capturing the discontinuities associated with the presence of the sharp crease through the use of coupled radial near-field and far-field solutions.

The main purpose of the present contribution is to revisit the wrinkling instability in its simplest form for the entire range of stiffness ratios. This will provide a clear framework in which to study the nonlinear behaviour of the system. A secondary goal is to identify the role of additional effects that may contribute to the instability. Indeed, in complicated mechanical and biological situations, it is not clear *a priori* that additional effects may be neglected and whether other instabilities may emerge. Here, we study—separately—the contributions of added surface tension, pressure, fibres, or the presence of an additional top layer. Our strategy is to offer a detailed description of the regular case in two extreme situations: firstly, the case where the two layers are equally strained during loading; and secondly, when the top layer grows and the bottom layer remains unstrained until the bifurcation. Then, we consider more briefly the role of supplementary effects on the two main limits for small and large wavelengths. Finally, we note that some of the results presented here are already contained in various forms in previous papers, but we include them here for completeness so that this paper can be used as a basis to explore the behaviour of solutions after the bifurcation in subsequent work.

## 2. The model

### (a) General formulation

**Figure 1.**
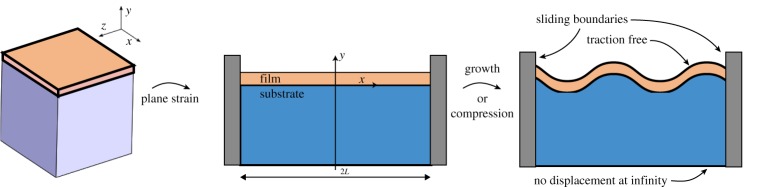
Geometry of the domain. The system is composed of a bilayer with an infinitely deep layer of width 2*L* bonded by a film of thickness 1. Considering only plane strain, the problem is reduced to the deformation of a two-dimensional system under either compression or growth causing wrinkling. The boundary conditions are: continuity of traction and displacement between the layers, sliding vertical boundaries with no detachment, traction free upper layer and no displacement or detachment at infinity. (Online version in colour.)

The basis of our computations is a three-dimensional formulation similar to those presented in [11,36]. We can substantially simplify the problem by only considering two-dimensional deformations, which is achieved by assuming that the material is in *plane strain*—that there is both no displacement in the transverse dimension and no dependence of the other components of the displacement on the spatial coordinate in that dimension. We consider the following model, illustrated in figure 1: let the region $B_{s}$ represent the initial unstressed infinite elastic substrate and $B_{f}$ be an elastic film bonded to its upper surface. Together, these form the domain $B=B_{f}\cup B_{s}$. Let *μ*_s_ and *μ*_f_ represent the shear moduli of their respective layers, *β* = *μ*_f_/*μ*_s_ be their ratio and **X** be a coordinate system across the two layers in the reference configuration. Let us henceforth fix our domains as $B_{f}=[-L,L]\times (0,1]$ (taking the thickness of the film to be 1 without loss of generality) and $B_{s}=[-L,L]\times (-\infty ,0]$ for some fixed *L* > 0 to be determined. After a static deformation, the new material coordinates of the deformed configuration are given by **x**(**X**) with deformation gradient

2.1 $$F=\frac{\partial x}{\partial X}.$$

We consider the two extreme cases that we label *growth* and *compression*.

### (b) Growth

The fundamental assumption which allows us to incorporate material growth into the framework of elasticity comes from the theory of morphoelasticity, as described in [37]. We assume that any residual stresses within the material in the absence of applied loads are the result of growth on a local level and hence that we can decompose the deformation gradient multiplicatively as

2.2 F=AG,

where **A** is an elastic deformation tensor and **G** is a growth tensor describing the local effects of growth. The application of the growth tensor alone to the reference configuration may not produce a physically realisable body, but the following application of the elastic tensor introduces stresses that enforce the boundary conditions and remove unphysical phenomena such as self-intersection. If we assume that our material is incompressible, we impose the constraint detA=1 almost everywhere—the only local changes in volume of the material come from the growth process.

For a hyperelastic material, with elastic strain-energy density function *W*, we can define an augmented energy density functional for the composed deformation by (see [38])

2.3 $$\bar{W}(F,G)=(\det G)W({FG}^{-1})-p\left(\det({FG}^{-1})-1\right).$$

Here, *p* is a Lagrange multiplier that imposes the incompressibility constraint. In the particular case of a neo-Hookean material that we use here, the strain-energy density is given by

2.4 $$W(A)=\frac{\mu }{2}(\text{tr}\;AA^{T}-3).$$

Let ***Ξ*** be the set of *admissible elastic deformations*—those deformations with sufficient regularity for (2.3) to be integrable. For a fixed growth tensor **G**, we can write the elastic deformation tensor **A** induced by any **x**∈***Ξ*** as

2.5 $$A={FG}^{-1}=\frac{\partial x}{\partial X}G^{-1}.$$

### (c) Growth and compression

We can also consider an additional lateral compression in addition to or in place of growth in our system. As with growth, we can specify an initial diagonal stretch tensor **A**_0_ to prescribe the external stretches that are applied to the bilayer. Since no new material is generated in this process, we must have $\det A_{0}=1$. Our multiplicative decomposition is now

2.6 $$F=AA_{0}G.$$

Since **A**_0_ represents an elastic process, our energy density functional (2.3) is unchanged. Indeed, we only separate it from **A** for notational convenience.

We will study initial stretch tensors **A**_0_ given by

2.7 $$A_{0}=\left(\begin{array}{cc} \lambda & 0\\ 0 & \lambda^{-1} \end{array}\right),$$

and a growth tensor **G** satisfying detG=J. In our case, we fix **G** = *g***I**, where

2.8 $$g(X)=\left\{ \begin{array}{ll} \gamma & X\in B_{f}\\ 1 & X\in B_{s} \end{array}\right..$$

We then have *J* = *g*^2^. In our study, we will specialize to the compression-only case by taking *J*=1.

We can now specify our mathematical problem. We look for deformations **x** that are local minimizers of the total elastic energy of our system, subject to the elastic incompressibility constraint detA=1. More explicitly, given **G** and **A**_0_, our variational problem is

2.9 $$\underset{\begin{array}{c} x\in \Xi \\ p\in \Lambda \end{array}}{\mathrm{minimize}}\;I(x,p):=\int_{B}\left(\frac{\mu }{2}\det G\right)\left(\text{tr}\left[AA^{T}A_{0}^{2}\right]-3\right)-p(\det A-1)\;dX,$$

where ***Λ*** is a suitable Lagrange multiplier space that allows us to impose the pointwise constraint on **A**. The Euler–Lagrange equation for this system yields a necessary condition on minimizers of the energy.

### (d) Stream functions

Taking advantage of the two-dimensional nature of the problem, we can make use of a technical tool to automatically satisfy the elastic incompressibility constraint. Essentially, we can find a *stream function* for the deformation, which is named after a similar construction used for the two-dimensional Stokes flow. The difference here is that the domain of the stream function is a *mixed coordinate space*—it is a function of coordinates in both the reference and deformed configurations. The idea was first proposed in this setting by Rooney & Carroll [39] and used in [11,40].

Let **x**(**X**) = (*x*(*X*, *Y* ), *y*(*X*, *Y* )) be any two-dimensional deformation for which

2.10 $$\det F=\frac{\partial x}{\partial X}\frac{\partial y}{\partial Y}-\frac{\partial x}{\partial Y}\frac{\partial y}{\partial X}\equiv J,$$

where *J* is constant in each subdomain of B. More general growth conditions can be incorporated into this formulation, but for simplicity we will only study the constant case. Any such **F** can be decomposed multiplicatively as in (2.2). Away from some pathological cases, we can use an implicit function theorem based argument to define a function ***Ψ*** on the mixed coordinates (*x*, *Y* ) such that

2.11 $$X=\frac{1}{J\lambda }\frac{\partial \Psi }{\partial Y}(x,Y)\;\mathrm{and}\;y=\frac{1}{\lambda }\frac{\partial \Psi }{\partial x}(x,Y).$$

From these representations, we can also compute the partial derivatives found in $F=\frac{\partial x}{\partial X}$ to rewrite the deformation gradient as

2.12 $$F=\frac{1}{\partial_{xY}\Psi }\left(\begin{array}{cc} J\lambda & -\partial_{YY}\Psi \\ J\partial_{xx}\Psi & \lambda^{-1}\left({\left(\partial_{xY}\Psi \right)}^{2}-\partial_{YY}\Psi \partial_{xx}\Psi \right) \end{array}\right).$$

Explicitly computing the determinant of **F**, we find

2.13 $$\det F=\frac{1}{{\left(\partial_{xY}\Psi \right)}^{2}}\left[J\left({\left(\partial_{xY}\Psi \right)}^{2}-\partial_{YY}\Psi \partial_{xx}\Psi \right)+J\partial_{xx}\Psi \partial_{YY}\Psi \right]=J,$$

hence the determinant constraint (2.10) is automatically satisfied exactly. To translate our energy functional into this stream function formulation, we make the change of integration variables

2.14 $$dxdY=\frac{\partial x}{\partial X}dXdY=\frac{J\lambda }{\partial_{xY}\Psi }dXdY.$$

Since detA=1 by construction, the Lagrange multiplier term in (2.9) disappears and leaves us with the minimization problem

2.15 $$\begin{array}{rl} \underset{\Psi \in \Phi }{\mathrm{minimize}}\;\tilde{I}(\Psi ) & \;:=\int_{\tilde{B}}\frac{\mu }{2J\lambda^{3}\partial_{xY}\Psi }[-2J\lambda^{2}{\left(\partial_{xY}\Psi \right)}^{2}+{\left(\partial_{xY}\Psi \right)}^{4}-2\partial_{YY}\Psi {\left(\partial_{xY}\Psi \right)}^{2}\partial_{xx}\Psi \\ \; & \;+J^{2}\lambda^{2}\left(\lambda^{2}+{\left(\partial_{xx}\Psi \right)}^{2}\right)+{\left(\partial_{YY}\Psi \right)}^{2}\left(\lambda^{2}+{\left(\partial_{xx}\Psi \right)}^{2}\right)]\;dx\;dY, \end{array}$$

where ***Φ*** is the set of admissible stream functions.

To obtain the Euler–Lagrange equation of (2.15) and its boundary conditions explicitly, we must compute the first variation of its integral functional $\tilde{I}$. For notational simplicity, we rewrite $\tilde{I}$ as

2.16 $$\tilde{I}(\Psi )=\int_{\tilde{B}}f(\partial_{xx}\Psi ,\partial_{xY}\Psi ,\partial_{YY}\Psi )\;dx\;dY.$$

The Euler–Lagrange equations for the system are then given by

2.17 $$\begin{array}{ll} & \frac{\partial^{2}}{\partial x^{2}}\left(\frac{\partial f}{\partial (\partial_{xx}\Psi_{f})}\right)+\frac{\partial^{2}}{\partial x\partial Y}\left(\frac{\partial f}{\partial (\partial_{xY}\Psi_{f})}\right)+\frac{\partial^{2}}{\partial Y^{2}}\left(\frac{\partial f}{\partial (\partial_{YY}\Psi_{f})}\right)=0\\ \mathrm{and}\; & \frac{\partial^{2}}{\partial x^{2}}\left(\frac{\partial f}{\partial (\partial_{xx}\Psi_{s})}\right)+\frac{\partial^{2}}{\partial x\partial Y}\left(\frac{\partial f}{\partial (\partial_{xY}\Psi_{s})}\right)+\frac{\partial^{2}}{\partial Y^{2}}\left(\frac{\partial f}{\partial (\partial_{YY}\Psi_{s})}\right)=0. \end{array}\}$$

### (e) Boundary condition

The physical constraints we impose on the system at the boundaries are illustrated in figure 1. We impose that all displacements in the substrate vanish at infinity, that the material may slide along—but not penetrate—the vertical sides of the domain and that the upper surface of the film is traction free. We define separate stream functions ***Ψ***_f_ and ***Ψ***_s_ for each layer of the system and we seek to simultaneously solve for the energy-minimizing stream function of each layer. The problems for each layer are coupled by the introduction of boundary conditions at the layer interfaces that impose continuity of traction and displacement between layers.

From the physical condition that the two layers cannot detach from one another, we obtain, at *Y* = 0:

2.18*a* $$\begin{array}{rl} & \frac{\partial \Psi_{f}}{\partial x}=\frac{\partial \Psi_{s}}{\partial x}, \end{array}$$

2.18*b* $$\begin{array}{rl} & \frac{\partial \Psi_{f}}{\partial Y}=\gamma^{2}\frac{\partial \Psi_{s}}{\partial Y}. \end{array}$$

From repeated integration by parts in our calculation of the first variation, we obtain additional natural boundary conditions at the interface (*Y* = 0), representing the physical conditions on the continuity of traction:

2.19*a* $$\begin{array}{rl} & \frac{\partial }{\partial Y}\left(\frac{\partial f}{\partial (\partial_{YY}\Psi_{f})}\right)+\frac{\partial }{\partial x}\left(\frac{\partial f}{\partial (\partial_{xY}\Psi_{f})}\right)=\frac{\partial }{\partial Y}\left(\frac{\partial f}{\partial (\partial_{YY}\Psi_{s})}\right)+\frac{\partial }{\partial x}\left(\frac{\partial f}{\partial (\partial_{xY}\Psi_{s})}\right), \end{array}$$

2.19*b* $$\begin{array}{rl} & \gamma^{2}\frac{\partial f}{\partial (\partial_{YY}\Psi_{f})}=\frac{\partial f}{\partial (\partial_{YY}\Psi_{s})}. \end{array}$$

On top of film (*Y* = 1), we obtain the traction-free conditions through the same process:

2.20*a* $$\begin{array}{rl} & \frac{\partial }{\partial Y}\left(\frac{\partial f}{\partial (\partial_{YY}\Psi_{f})}\right)+\frac{\partial }{\partial x}\left(\frac{\partial f}{\partial (\partial_{xY}\Psi_{f})}\right)=0, \end{array}$$

2.20*b* $$\begin{array}{rl} & \frac{\partial f}{\partial (\partial_{YY}\Psi_{f})}=0. \end{array}$$

Finally, we impose the decay conditions

2.21*a* $$\begin{array}{rl} & \;\lim_{Y\to -\infty }\partial_{x}\Psi_{s}-Y=0, \end{array}$$

2.21*b* $$\begin{array}{rl} & \;\lim_{Y\to -\infty }\partial_{Y}\Psi_{s}-x=0. \end{array}$$

The two fourth-order PDEs for ***Ψ***_f_ and ***Ψ***_s_ in (2.17) and the eight boundary conditions given by (2.18)–(2.21) form the full Euler–Lagrange system. It should be noted that the explicit form of these Euler–Lagrange equations and their boundary conditions are lengthy with significant nonlinearity, making their direct solution impossible through analytic means.

### (f) Perturbation

Despite the difficulties that a complete characterization of solutions to this problem presents, it is easy to see that the homogeneous growth solution given by

2.22 $$x^{\left(0\right)}(X,Y)=\left\{ \begin{array}{ll} \left(\lambda X,\gamma^{2}\lambda^{-1}Y\right) & (X,Y)\in B_{f},\\ \left(\lambda X,\lambda^{-1}Y\right) & (X,Y)\in B_{s}, \end{array}\right.$$

with corresponding stream functions

2.23 $$\begin{array}{ll} & \Psi_{f}^{\left(0\right)}(x,Y)=\gamma^{2}xY\;Y\in (0,1]\\ \mathrm{and} & \Psi_{s}^{\left(0\right)}(x,Y)=xY\;Y\in (-\infty ,0], \end{array}\}$$

is a solution of (2.17). Consider a perturbation of the form ***Ψ*** = ***Ψ***^(0)^ + *ϵ**Ψ***^(1)^, where *ϵ* is a small positive parameter. To linear order in *ϵ*, the Euler–Lagrange equations for the system read

2.24 $$\begin{array}{ll} & \lambda^{2}\frac{\partial^{4}\Psi_{f}^{\left(1\right)}}{\partial Y^{4}}+(\gamma^{4}+\lambda^{4})\frac{\partial^{4}\Psi_{f}^{\left(1\right)}}{\partial x^{2}\partial Y^{2}}+\gamma^{4}\lambda^{2}\frac{\partial^{4}\Psi_{f}^{\left(1\right)}}{\partial x^{4}}=0\\ \mathrm{and} & \lambda^{2}\frac{\partial^{4}\Psi_{s}^{\left(1\right)}}{\partial Y^{4}}+(1+\lambda^{4})\frac{\partial^{4}\Psi_{s}^{\left(1\right)}}{\partial x^{2}\partial Y^{2}}+\lambda^{2}\frac{\partial^{4}\Psi_{s}^{\left(1\right)}}{\partial x^{4}}=0, \end{array}\}$$

with boundary conditions given explicitly in appendix A. Assuming a periodic decomposition of the form ***Ψ***^(1)^(*x*, *Y* ) = sin(*kx*)*h*^(1)^(*Y* ) for some *k* > 0, we arrive at the ODEs

2.25 $$\begin{array}{ll} & \lambda^{2}\frac{d^{4}h_{f}^{\left(1\right)}}{dY^{4}}-k^{2}(\gamma^{4}+\lambda^{4})\frac{d^{2}h_{f}^{\left(1\right)}}{dY^{2}}+\gamma^{4}k^{4}\lambda^{2}h_{f}^{\left(1\right)}=0\\ \mathrm{and}\; & \lambda^{2}\frac{d^{4}h_{s}^{\left(1\right)}}{dY^{4}}-k^{2}(1+\lambda^{4})\frac{d^{2}h_{s}^{\left(1\right)}}{dY^{2}}+k^{4}\lambda^{2}h_{s}^{\left(1\right)}=0, \end{array}\}$$

with boundary conditions given in appendix A. Solving (2.25) with the decay conditions at *Y* → ∞ (A 9g–A 9h), we obtain the general solutions

2.26 $$\begin{array}{ll} & h_{f}^{\left(1\right)}(Y)=c_{1}e^{-k\gamma^{2}Y}+c_{2}e^{k\gamma^{2}Y}+c_{3}e^{-kY}+c_{4}e^{kY}\\ \mathrm{and}\; & h_{s}^{\left(1\right)}(Y)=c_{5}e^{kY}+c_{6}Ye^{kY}, \end{array}\}$$

in the case λ = 1,

2.27 $$\begin{array}{ll} & h_{f}^{\left(1\right)}(Y)=c_{1}e^{-k\lambda Y}+c_{2}Ye^{-k\lambda Y}+c_{3}e^{k\lambda Y}+c_{4}Ye^{k\lambda Y}\\ \mathrm{and}\; & h_{s}^{\left(1\right)}(Y)=c_{5}e^{k\lambda^{-1}Y}+c_{6}e^{k\lambda Y}, \end{array}\}$$

in the case λ = *γ* and

2.28 $$\begin{array}{ll} & h_{f}^{\left(1\right)}(Y)=c_{1}e^{-k\gamma^{2}\lambda^{-1}Y}+c_{2}e^{k\gamma^{2}\lambda^{-1}Y}+c_{3}e^{-k\lambda Y}+c_{4}e^{k\lambda Y}\\ \mathrm{and}\; & h_{s}^{\left(1\right)}(Y)=c_{5}e^{k\lambda^{-1}Y}+c_{6}e^{k\lambda Y}, \end{array}\}$$

otherwise. Substituting these expressions into our boundary conditions, we obtain a homogeneous system of six linear equations in the six unknown coefficients **c**: ={*c*_*i*_}^6^_*i*=1_ that can be abbreviated as

2.29 M(k,γ,β)c=0,

where **M** is a 6 × 6 matrix. This system will only have non-trivial solutions if detM=0, thus giving us a solvability condition for our system.

## 3. Linear analysis

We now focus our attention on two specific cases: a bilayer that is compressed unilaterally but experiences no growth and a bilayer that is under no compression but has a growing upper layer. The determinant of **M** is sufficiently complex that its zero level set cannot be obtained in closed form. However, it can be obtained asymptotically for short and long wavelengths and solved numerically in the intermediate regime.

### (a) Compression

In the case of pure compression, we set *γ* = 1 and consider λ as our bifurcation parameter. The determinant of **M** can be written in the form

3.1 $$\det M(k,\lambda ,\beta )=\frac{1}{\lambda^{7}}\sum_{i=0}^{4}p_{i}(k,\lambda ,\beta )e^{k\zeta_{i}},$$

where each *p*_*i*_ is a polynomial in its arguments and (*ζ*_*i*_)^4^_*i*=0_ = (0, λ^−1^ + λ,  − λ^−1^ + λ, λ^−1^ − λ,  − λ^−1^ − λ). For large values of *k*, $\exp(k\zeta_{1})$ is the dominant term and thus, *p*_1_ must vanish in order for the determinant to vanish in that limit. This polynomial—which has total degree 34—vanishes whenever λ is equal to either a particular root of the equation

3.2 $$\lambda^{3}+\lambda^{2}+\lambda -1=0,$$

given by

3.3 $$\lambda_{\mathrm{biot}}=\frac{1}{3}\left({\left(17+3\sqrt{33}\right)}^{1/3}-\frac{2}{{\left(17+3\sqrt{33}\right)}^{1/3}}-1\right)\approx 0.543689,$$

or a particular root λ_*_(*β*) of a polynomial given by the equation

3.4 $$\begin{array}{ll} (1+\beta )\lambda^{3}+(1-\beta )\lambda^{2}+(1+\beta )\lambda -1+\beta =0 & \mathrm{if}\;\beta <1,\\ (1+\beta )\lambda^{3}-(1-\beta )\lambda^{2}+(1+\beta )\lambda +1-\beta =0 & \mathrm{if}\;\beta >1. \end{array}\}$$

The root in question is not present in the case *β* = 1 (this is in fact the classical Biot instability of an elastic half space as the two layers can no longer be distinguished), but, when it exists, it is always strictly less than λ_biot_. Thus, λ_biot_ provides a lower bound for the critical compression ratio required to cause the emergence of non-trivial solutions.

**Figure 2.**
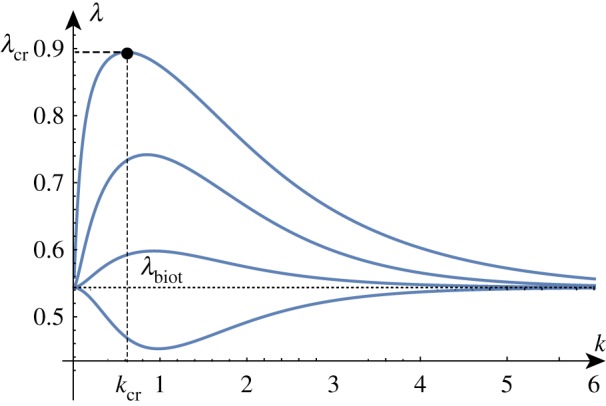
The maxima of the dispersion curves of det(**M**) = 0 in the λ-*k* plane provide the first critical values of λ at which oscillatory solutions can be obtained. For instance, the critical compression λ_cr_ and wavenumber *k*_cr_ are indicated for the top curve. The curves from top to bottom are obtained for decreasing values of *β*∈{10, 5/2, 5/4, 5/16}. (Online version in colour.)

**Figure 3.**
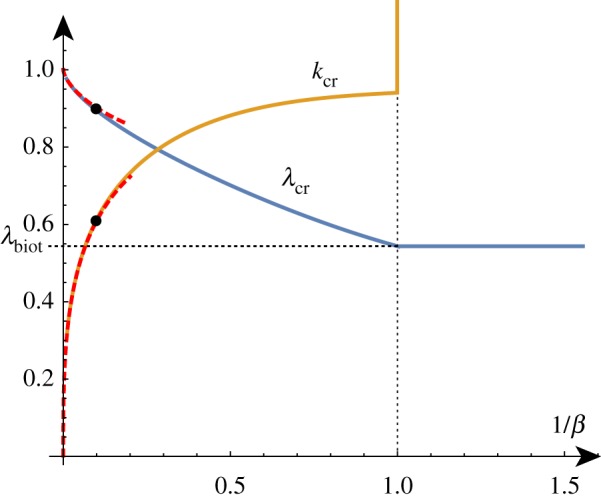
The critical wavenumber *k*_cr_ and compression λ_cr_ as functions of *β*^−1^. The two points on the curves correspond to the critical values found for *β* = 10. For *β* < 1, the critical compression is given by the Biot value and *k*_cr_ → ∞. The asymptotic estimates provide good approximations of these curves up to *β* ≈ 20 (red/dashed). Please note that the vertical axis in this figure and similar figures found later in the paper represents *both* the wavenumber and the compression as indicated by the curve labels. (Online version in colour.)

To better understand the solution set, we can solve the determinant relation numerically. We can fix a stiffness ratio *β* and find the compression ratio λ as a function of the wavenumber *k*. An example of such a *dispersion curve* is shown in figure 2 for the particular value *β* = 10. From this, we can deduce that if we were to gradually decrease the compression ratio λ from 1, we would expect to see non-trivial periodic solutions emerging at λ_cr_ ≈ 0.89 with wavenumber *k*_cr_ ≈ 0.61. We can repeat this process and track the position of this critical point as we vary the value of *β*, as shown in figure 3. As *β* decreases towards 1, λ_cr_ approaches λ_biot_. For values of *β* infinitesimally above 1, a finite wavenumber *k* ≈ 0.941 is selected, but at *β* = 1, all wavenumbers are possible. For *β* < 1, we see the reappearance of a critical point, but it is in fact a local minimum rather than a local maximum. Hence, surface instability appears first for all values of *β* < 1.

For large values of *β* corresponding to a stiff film on a soft substrate, the selected wavenumber becomes vanishingly small and the critical compression ratio on an infinite domain approaches 1, corresponding to the Euler buckling instability. A standard asymptotic analysis reveals the following approximations (illustrated in figure 3):

3.5 $$\begin{array}{rl} \lambda_{\mathrm{cr}} & \;=1-\frac{3^{2/3}}{4}\beta^{-2/3}+\frac{33\cdot 3^{1/3}}{160}\beta^{-4/3}-\frac{3^{2/3}}{8}\beta^{-5/3}\\ & \;\;-\frac{7629}{22400}\beta^{-2}+\frac{39\cdot 3^{1/3}}{160}\beta^{-7/3}+\frac{3302617}{5376000\cdot 3^{1/3}}\beta^{-8/3}+O(\beta^{-3}), \end{array}$$

3.6 $$\begin{array}{rl} k_{\mathrm{cr}} & \;=3^{1/3}\beta^{-1/3}-\frac{3}{5}\beta^{-1}+\frac{463\cdot 3^{2/3}}{5600}\beta^{-5/3}+\frac{3217}{33600\cdot 3^{2/3}}\beta^{-7/3}+O(\beta^{-8/3}). \end{array}$$

We recover the well-known dependence for the wavelength with a *β*^1/3^ scaling that was already established by Biot [41] and has been recovered numerous times since then (see [42], for example).

### (b) Growth

The growth case displays many similarities to the compression case. Considering large values of *k* once more reveals the existence of a Biot-type wrinkling instability for the system as described in figure 1. As before, the determinant can be written in the form

3.7 $$\det M(k,\gamma ,\beta )=\frac{1}{\gamma^{3}}\sum_{i=0}^{4}p_{i}(k,\gamma ,\beta )e^{k\zeta_{i}},$$

where each *p*_*i*_ is a polynomial in its arguments and (*ζ*_*i*_)^4^_*i*=0_ = (0, 1 + *γ*^2^,  − 1 + *γ*^2^,  − 1 − *γ*^2^, 1 − *γ*^2^). For large enough *k*, $\exp(k\zeta_{1})$ is the dominant term and hence in order for the determinant to vanish in that limit, *p*_1_ must vanish. We find that polynomial *p*_1_ vanishes whenever *γ* is equal to either a particular root of the equation

3.8 $$\gamma^{3}-\gamma^{2}-\gamma -1=0,$$

given by

3.9 $$\gamma_{\mathrm{biot}}=\frac{1}{\lambda_{\mathrm{biot}}}=\frac{1}{3}\left(1+{\left(19-3\sqrt{33}\right)}^{\frac{1}{3}}+{\left(19+3\sqrt{33}\right)}^{\frac{1}{3}}\right)\approx 1.83929,$$

or a particular root γ_*_(*β*) of the equation

3.10 $$\beta^{2}\gamma^{6}-(3\beta^{2}+2\beta )\gamma^{4}-(\beta^{2}+4\beta +4)\gamma^{2}-(\beta^{2}+2\beta )=0.$$

Further examination reveals that we have γ_*_(*β*) > *γ*_biot_ for all values of *β* > 0. Thus, *γ*_biot_ provides an upper bound on the critical growth factor required in order to achieve non-trivial periodic solutions.

**Figure 4.**
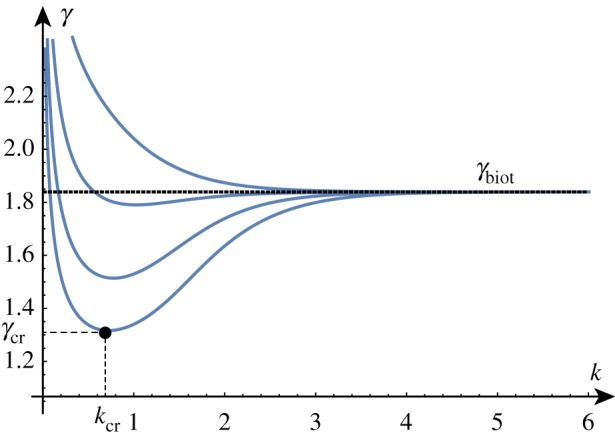
Solutions of the dispersion relation for a range of *β* values. In a thought experiment, the film grows starting at *γ* = 1. The homogeneous solution remains stable until a new solution emerges at *γ* = *γ*_cr_ associated with wavenumber *k* = *k*_cr_. For *β* > *β*_min_, the solution arises before Biot's instability (indicated by a dashed line). The two upper curves are obtained for values of *β*∈ {5/2, 5/4, 5/8, 5/16} just above and just below the critical value *β*_min_. (Online version in colour.)

Solving the determinant relation numerically once more, we can fix a stiffness ratio *β* and find the growth factor *γ* as a function of the wavenumber *k*. Examples of such dispersion relations are shown in figure 4.

**Figure 5.**
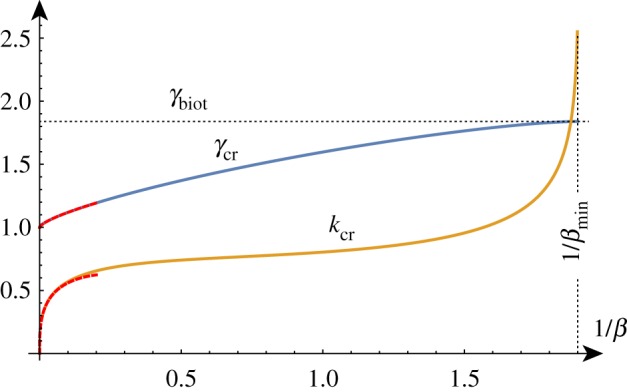
Critical solutions value of growth and wavenumber as a function of 1/*β* (asymptotic approximations shown dashed). Critical solutions exist for *β* > *β*_min_ ≈ 1/1.9, after which, the Biot instability is the dominant instability. (Online version in colour.)

In a thought experiment where we gradually increase the growth factor *γ* from 1, we expect to see nontrivial periodic solutions emerging at *γ*_cr_. As before, by repeating this process, we can track the position of this critical point as we vary the value of *β*, as shown in figure 5. As *β* decreases, *γ*_cr_ approaches *γ*_biot_ and we see that the value of *k*_cr_ increases without bound, demonstrating the aforementioned instability. The value of *β* = *β*_min_ at which the wavenumber first diverges can be found exactly (but is not given analytically here) and is *β*_min_ ≈ 1.90379.

As shown in figure 5, for large values of *β*, the critical values are well approximated by

3.11 $$\begin{array}{rl} \gamma_{\mathrm{cr}}^{2} & \;=1+\frac{3^{2/3}}{2}\beta^{-2/3}+\frac{2\cdot 3^{1/3}}{5}\beta^{-4/3}+\frac{201}{2800}\beta^{-2}-\frac{27403}{112000\cdot 3^{1/3}}\beta^{-8/3}\\ & \;\;+\frac{583461\cdot 3^{1/3}}{21560000}\beta^{-10/3}-\frac{553132947}{22422400000}\beta^{-4}+O(\beta^{-14/3}) \end{array}$$

3.12 $$\begin{array}{rl} k_{\mathrm{cr}} & \;=3^{1/3}\beta^{-1/3}-\frac{11}{10}\beta^{-1}+\frac{881}{1400\cdot 3^{1/3}}\beta^{-5/3}+\frac{601}{2800\times 3^{2/3}}\beta^{-7/3}\\ & \;\;-\frac{1193837}{8624000}\beta^{-3}+\frac{56746499}{343200000\cdot 3^{1/3}}\beta^{-11/3}+O(\beta^{-13/3}). \end{array}$$

## 4. Generalizations

We now investigate a number of modifications to the physical problem that model different effects seen in nature. Of particular interest is the effect of these changes on the presence and position of Euler-type (large wavelength) and Biot-type (small wavelength) instabilities in the system. To this end, we repeat the linear analysis found in figure 3, adding additional insights where necessary. Since the method has already been described at length, we briefly explain the new aspects of the problem without details.

### (a) Bilayer with surface tension

The first modification we consider is the addition of surface energy. In elastic solids, there is an energetic cost to maintaining a surface that we must incorporate into our variational formulation when the material is sufficiently soft or to model the effect of a small layer on top of the material surface. To do this, we add another term to the energy functional in (2.9) to represent the surface energy at the interface between the layers and/or at the top of the upper layer. Following [11], this contribution takes the form

4.1 $$d\int_{\Gamma }\;ds,$$

where *d* is a surface energy density and *Γ* is a subset of $\partial B_{f}\cup \partial B_{s}$. The addition of this term has no effect on the bulk Euler–Lagrange equations (2.17), but instead modifies the boundary conditions. In particular, (A 9a) becomes

4.2 $$\lambda^{2}\frac{d^{3}h_{f}^{\left(1\right)}}{dY^{3}}(1)-k^{2}(2\gamma^{4}+\lambda^{4})\frac{dh_{f}^{\left(1\right)}}{dY}(1)-d\beta^{-1}k^{4}\gamma^{6}\lambda h_{f}^{\left(1\right)}(1)=0.$$

This extra term adds dependence on the surface energy parameter *d* to the system of linear equations (2.29) so that it is now of the form

4.3 $$\hat{M}(k,\gamma ,\lambda ,\beta ,d)c=0.$$

As before, this homogeneous system of linear equations has non-trivial solutions precisely when the determinant of the matrix $\hat{M}$ vanishes.

#### (i) Compression

In the compression case (*γ* = 1), we can write the determinant in the form

**Figure 6.**
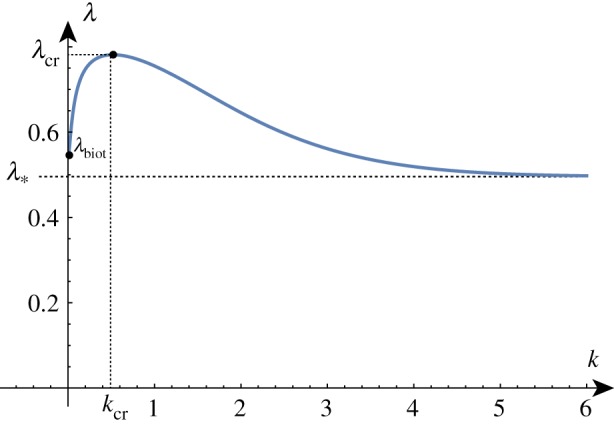
λ as a function of *k* for *β* = 10, *d* = 7.5 (here *k*_cr_ ≈ 0.58 and λ_cr_ ≈ 0.78). (Online version in colour.)

4.4 $$\det\hat{M}(k,\lambda ,\beta ,d)=\frac{1}{\lambda^{5}}\sum_{i=0}^{4}{\hat{p}}_{i}(k,\lambda ,\beta ,d)e^{k\zeta_{i}},$$

where each ${\hat{p}}_{i}$ is some polynomial in its arguments and (*ζ*_*i*_)^4^_*i*=0_ = (0, λ^−1^ + λ,  − λ^−1^ + λ, λ^−1^ − λ,  − λ^−1^ − λ). First, we remark that *k* = 0 is always a solution for λ = λ_biot_. Second, for large values of *k*, we have again that $\exp(k\zeta_{1})$ is the dominant term and hence for large *k*, ${\hat{p}}_{1}$ must vanish. There is no longer a zero of this polynomial at λ_biot_ for all *k*, but there is still one at λ_*_(*β*) (for *β*≠1). For this root, we have λ_*_(*β*) < λ_biot_ for all *β* > 0 as shown in figure 6. Hence, we conclude that as λ decreases, it eventually reaches λ_biot_ at *k* = 0 which becomes the first instability.

**Figure 7.**
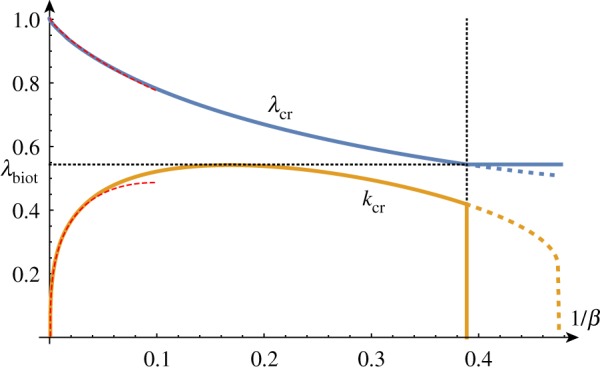
Exact (solid) and approximate (dashed) values of *k*_cr_ and λ_cr_ as functions of *β*^−1^ for *d* = 7.5. (Online version in colour.)

When we compute the position of the critical growth and wavenumber as a function of *β*^−1^ (plotted in figure 7), we see a dramatic change in the qualitative behaviour of both quantities. Firstly, we see the disappearance of the critical point for values of *β*≲2.1. However, the critical point ceases to be a global maximum before this occurs: for values of *β*≲2.6 the global maximum of the dispersion curve occurs at *k* = 0 with a selected compression ratio of λ_biot_. Hence, the addition of surface tension prevents the Biot instability from occurring, replacing it with a Euler-type instability: if the film is sufficiently soft then the whole system buckles in a similar manner to a beam instead of displaying periodic fine wrinkling.

**Figure 8.**
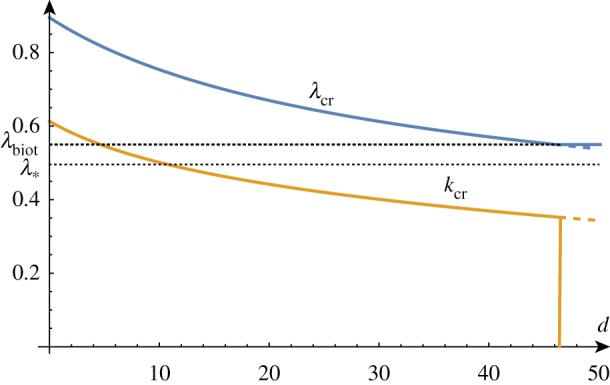
*k*_cr_ and λ_cr_ as functions of *d* for *β* = 10. (Online version in colour.)

With the addition of another parameter, we can also fix the value of *β* and track the change in the critical growth and wavenumber as *d* varies. As one might expect, figure 8 demonstrates that the higher the surface energy density, the lower the compression ratio required to induce wrinkling and the lower the wavenumber of the wrinkling.

As before, for large values of *β* corresponding to a stiff film on a soft substrate, the selected wavenumber becomes vanishingly small and the critical compression factor approaches 1. A standard asymptotic analysis reveals the following approximations (illustrated in figure 7) that demonstrate the influence of the surface energy parameter *d* on the critical growth factor and wavenumber selection when compared with (3.5) and (3.6):

4.5 $$\begin{array}{rl} \lambda_{\mathrm{cr}} & \;=1-\frac{3^{2/3}}{4}\beta^{-2/3}-\frac{d}{4}\beta^{-1}-\frac{33\cdot 3^{1/3}}{160}\beta^{-4/3}\\ & \;\;-\frac{1}{16}\left(2\cdot 3^{2/3}-3\cdot 3^{2/3}d\right)\beta^{-5/3}+O(\beta^{-2}), \end{array}$$

4.6 $$\begin{array}{rl} k_{\mathrm{cr}} & \;=3^{1/3}\beta^{-1/3}-\frac{3}{5}\beta^{-1}-\frac{1}{4}3^{1/3}d\beta^{-4/3}+O(\beta^{-5/3}). \end{array}$$

#### (ii) Growth

In the growth case, we can write the determinant in the form

4.7 $$\det\hat{M}(k,\gamma ,\beta ,d)=\frac{1}{\gamma^{3}}\sum_{i=0}^{4}{\hat{p}}_{i}(k,\gamma ,\beta ,d)e^{k\zeta_{i}},$$

where each ${\hat{p}}_{i}$ is some polynomial in its arguments and (*ζ*_*i*_)^4^_*i*=0_ = (0, 1 + *γ*^2^,  − 1 + *γ*^2^,  − 1 − *γ*^2^, 1 − *γ*^2^). Consideration of the dominant term in the large *k* limit yields an asymptote at *γ* = γ_*_(*β*), which approaches *γ*_biot_ from above in the large *β* limit. As in the compression case, this asymptote is independent from *d*. The dispersion curve is similar to figure 2 with γ_*_(*β*) replacing *γ*_biot_. Echoing the results from the compression case, the critical growth factor is significantly increased, but occurs at a smaller wavenumber.

**Figure 9.**
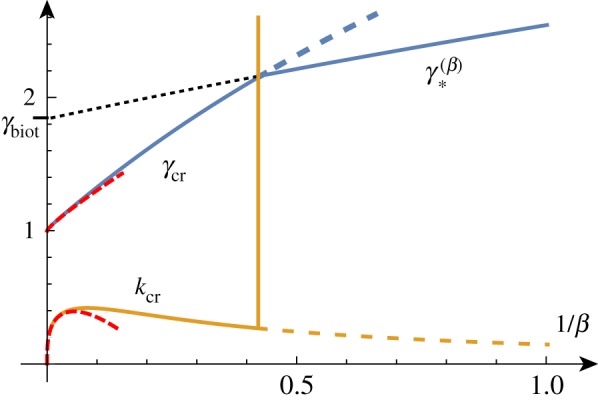
Exact (solid) and approximate (dashed) values of *k*_cr_ and *γ*_cr_ as functions of *β*^−1^ for *d* = 7.5. (Online version in colour.)

When we compute the position of the critical growth and wavenumber as a function of *β*^−1^ (plotted in figure 9), we see a dramatic change in the qualitative behaviour of both quantities. In particular, we no longer see a blow-up in the wavenumber as *β* decreases and we see an apparent increase in *γ*_cr_ without bound. However, the critical point that we are computing stops being a global minimum of *γ* for sufficiently small values of *β*. For *β* under this threshold, we would again expect a Biot-type instability.

**Figure 10.**
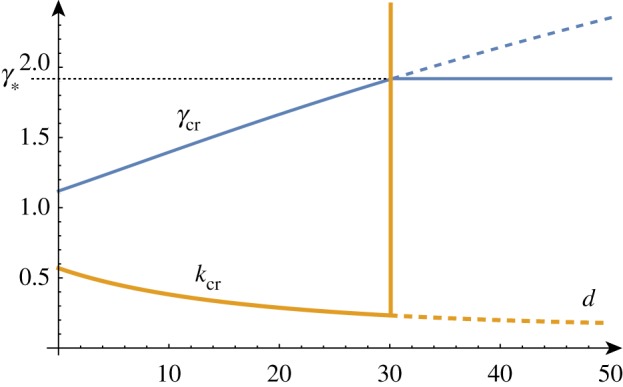
*k*_cr_ and *γ*_cr_ as functions of *d* for *β* = 10. (Online version in colour.)

Plotting the critical growth factor and wavenumber as a function of *d* (shown in figure 10) reveals that the higher the surface energy density, the higher the growth factor required to induce wrinkling and the lower the wavenumber of the wrinkling.

Another standard asymptotic analysis for large values of *β* gives the following approximations (illustrated in figure 9) for the correction that the surface energy parameter *d* induces on the critical growth factor:

4.8 $$\begin{array}{rl} \gamma_{\mathrm{cr}}^{2} & \;=1+\frac{3^{2/3}}{2}\beta^{-2/3}+\frac{d}{2}\beta^{-1}+\frac{2\cdot 3^{1/3}}{5}\beta^{-4/3}+\frac{5d}{8\cdot 3^{1/3}}\beta^{-5/3}+O(\beta^{-2}), \end{array}$$

4.9 $$\begin{array}{rl} k_{\mathrm{cr}} & \;=3^{1/3}\beta^{-1/3}-\frac{11}{10}\beta^{-1}-\frac{5d}{4\cdot 3^{2/3}}\beta^{-4/3}+O(\beta^{-5/3}). \end{array}$$

### (b) Bilayer with upper substrate

A second modification is to add another elastic layer (of either finite or infinite thickness) on top of the film. Here, we modify B to include an additional subdomain $B_{t}$ with shear modulus *μ*_t_ and relabel the stiffness ratios as *β*_f_: =*μ*_f_/*μ*_s_ and *β*_t_: =*μ*_t_/*μ*_s_. With $B_{t}=[-L,L]\times (0,\infty ]$, we now have a system of three ODEs for our Euler–Lagrange equations:

4.10 $$\begin{array}{ll} & \lambda^{2}\frac{d^{4}h_{t}^{\left(1\right)}}{dY^{4}}-k^{2}(1+\lambda^{4})\frac{d^{2}h_{t}^{\left(1\right)}}{dY^{2}}+k^{4}\lambda^{2}h_{t}^{\left(1\right)}=0,\\ & \lambda^{2}\frac{d^{4}h_{f}^{\left(1\right)}}{dY^{4}}-k^{2}(\gamma^{4}+\lambda^{4})\frac{d^{2}h_{f}^{\left(1\right)}}{dY^{2}}+\gamma^{4}k^{4}\lambda^{2}h_{f}^{\left(1\right)}=0\\ \mathrm{and}\;\; & \lambda^{2}\frac{d^{4}h_{s}^{\left(1\right)}}{dY^{4}}-k^{2}(1+\lambda^{4})\frac{d^{2}h_{s}^{\left(1\right)}}{dY^{2}}+k^{4}\lambda^{2}h_{s}^{\left(1\right)}=0, \end{array}\}$$

with boundary conditions given in appendix A. After consideration of the decay conditions at *Y* → + ∞ (A 10k) and (A 10l), we obtain the following general solution for *h*_t_ (the others are unchanged):

4.11 $$h_{t}(Y)=\left\{ \begin{array}{ll} c_{7}e^{-kY}+c_{8}Ye^{-kY} & \mathrm{if}\;\lambda =1,\\ c_{7}e^{-k\lambda^{-1}Y}+c_{8}e^{-k\lambda Y} & \mathrm{otherwise}. \end{array}\right.$$

Following the same method as before, we obtain a homogeneous linear system of eight equations in the eight unknowns $\tilde{c}:={\left\{c_{i}\right\}}_{i=1}^{8}$. This leads to the solvability condition

4.12 $$\tilde{M}(k,\gamma ,\lambda ,\beta_{f},\beta_{t})\tilde{c}=0,$$

which only has nontrivial solutions if $\det\tilde{M}=0$.

#### (i) Compression

In the compression case, on numerically plotting the solution set of the determinant relation, we find a similar dispersion curve compared to the unmodified problem (figure 2) but with λ_biot_ replaced by an asymptote λ_*_ that depends on both *β*_f_ and *β*_t_.

**Figure 11.**
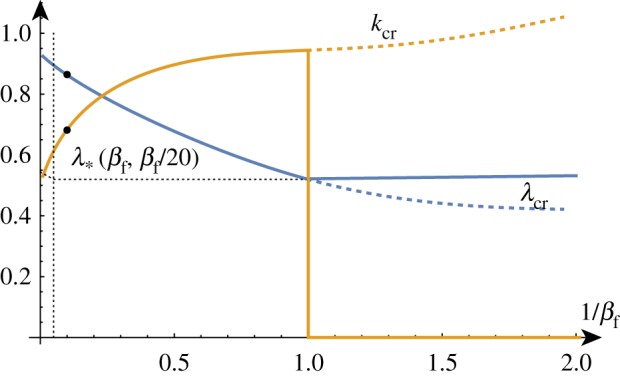
The critical wavenumber *k*_cr_ and compression λ_cr_ as functions of *β*^−1^_f_ for *β*_t_ = *β*_f_/20. (Online version in colour.)

The addition of another elastic layer decreases the critical compression ratio and the compression threshold for large *k* while increasing the critical wavenumber. For a given, fixed stiffness ratio *β*_f_*β*^−1^_t_, from figure 11 we can see that as *β*_f_ and *β*_t_ decrease, the critical compression ratio approaches the previously discussed threshold. However, we now find that for *β* < 1, we have a Euler-type buckling instability where the wavenumber *k* = 0 is selected.

**Figure 12.**
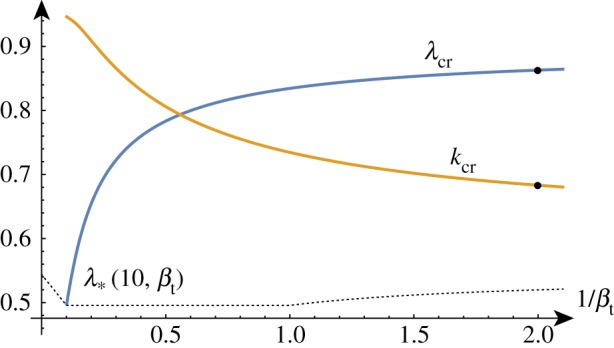
The critical wavenumber *k*_cr_ and compression λ_cr_ as functions of *β*^−1^_t_ for *β*_f_ = 10. (Online version in colour.)

By contrast, figure 12 shows us that if we fix *β*_f_ and vary *β*_t_, we observe a gradual increase in λ_cr_ and a gradual decrease in *k*_cr_ as *β*_t_ decreases, with the wavenumber remaining well determined. Thus, the addition of an upper layer decreases the critical compression ratio and increases the critical wavenumber selected in the system.

For films with large stiffness, the asymptotic expressions are

4.13 $$\begin{array}{rl} \lambda_{\mathrm{cr}} & \;=1-\frac{k_{1}^{3}+6\beta_{t}+6}{12\;k_{1}}\beta_{f}^{-2/3}+\frac{99\left(\beta_{t}+1\right){\;}^{2}}{160\;k_{1}^{2}}\beta_{f}^{-4/3}+O(\beta_{f}^{-2}), \end{array}$$

4.14 $$\begin{array}{rl} k_{\mathrm{cr}} & \;=k_{1}\beta_{f}^{-1/3}-\frac{3}{5}\left(\beta_{t}+1\right)+O(\beta_{f}^{-5/3}), \end{array}$$

where *k*_1_ = (3 + 3*β*_f_)^1/3^.

#### (ii) Growth

Repeating the techniques used in our previous cases, for large *k* we identify a critical growth threshold at a particular root *γ*_⋆_(*β*_f_, *β*_t_) of the following equation:

4.15 $$\begin{array}{ll} \beta_{f}^{2}\gamma^{6}-(3\beta_{f}^{2}+2\beta_{f}\beta_{t})\gamma^{4}-(\beta_{f}^{2}+4\beta_{f}\beta_{t}+4\beta_{t}^{2})\gamma^{2}-(\beta_{f}^{2}+2\beta_{f}\beta_{t})=0 & \beta_{t}<1,\\ \beta_{f}^{2}\gamma^{6}-(3\beta_{f}^{2}+2\beta_{f})\gamma^{4}-(\beta_{f}^{2}+4\beta_{f}+4)\gamma^{2}-(\beta_{f}^{2}+2\beta_{f})=0 & \beta_{t}\geq 1. \end{array}\}$$

**Figure 13.**
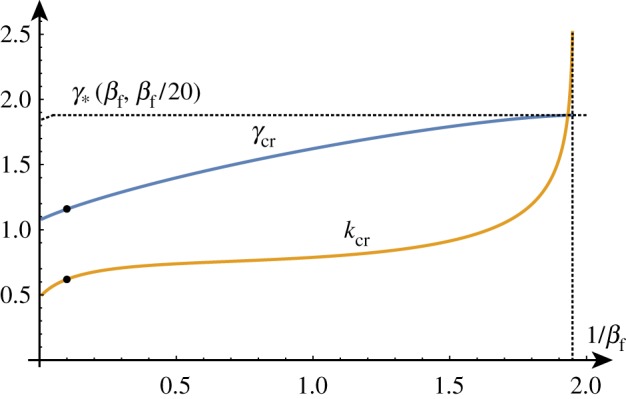
The critical wavenumber *k*_cr_ and compression λ_cr_ as functions of *β*^−1^_f_ for *β*_t_ = *β*_f_/20. (Online version in colour.)

Thus, we see that whichever substrate is softer dictates the position of the large *k* asymptote. In the limit of small *β*_t_ (very soft upper layer), we see that $\gamma_{⋆}\to \gamma_{\mathrm{biot}}$ is a solution of the relation as before and we recover the bilayer. As in the compression case, the profile of the dispersion curve is similar to the corresponding unmodified problem (figure 4). The addition of another elastic layer only slightly increases the critical growth factor, the critical wavenumber and the growth threshold for large *k* (shown in figure 13).

**Figure 14.**
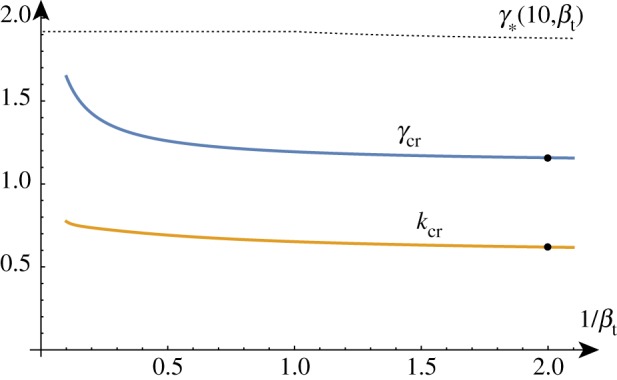
The critical wavenumber *k*_cr_ and compression λ_cr_ as functions of *β*^−1^_t_ for *β*_f_ = 10. (Online version in colour.)

Finally, figure 14 demonstrates that if we again fix *β*_f_ and decrease *β*_t_, *γ*_cr_ and *k*_cr_ both decrease with no apparent blow-up behaviour. Hence, the addition of an upper layer increases the critical growth factor and critical wavenumber selected in the system. In particular, as the stiffness of the upper layer approaches that of the film from below, the critical growth and wavenumber increase without bound.

### (c) Pressure

We can derive the effect of a normal pressure of magnitude *p* acting on top of the film layer by directly imposing this constraint on the surface. A pressure *p* on the surface can be expressed in terms of the Cauchy stress tensor **T** as **T** · **n** = *p***n** for all points on the top surface. To express this condition, we compute, to first order, the normal vector field and the Cauchy stress. First, we recall that for a sufficiently regular deformation, the unit surface normal vector in the deformed configuration is given by

4.16 $$n(X)=\frac{1}{\sqrt{{\left(\frac{\partial y}{\partial X}(X,1)\right)}^{2}+1}}\left(-\frac{\partial y}{\partial X}(X,1),1\right).$$

After changing coordinates into our stream function formulation, applying our perturbation ***Ψ*** = ***Ψ***^(0)^ + *ϵ**Ψ***^(1)^ from the homogeneous solution (2.22) and our periodic decomposition ***Ψ***^(1)^(*x*, *Y* ) = sin(*kx*)*h*^(1)^(*Y* ), we can rewrite (4.16) as:

4.17 $$n(x)=(0,1)+\left(1,\frac{1}{2}\right)k^{2}h(1)\sin(kx)\epsilon +O(\epsilon^{2}).$$

Second, we compute the Cauchy stress by using the constitutive equations for an incompressible neo-Hookean material [37, p. 286]

4.18 $$T=\mu AA^{T}-qI,$$

where *q* denotes the Lagrange multiplier associated with incompressibility. We expand both **T** = **T**^(0)^ + *ϵ***T**^(0)^ and *q* = *q*_0_ + *ϵq*_1_. Since the base solution is homogeneous, it can be solved directly by using the boundary condition and to order 0 in *ϵ*, we find

4.19 $$T^{\left(0\right)}=\left(\begin{array}{cc} \frac{\lambda^{2}\mu_{f}}{\gamma^{2}}-q_{0} & 0\\ 0 & \frac{\gamma^{2}\mu_{f}}{\lambda^{2}}-q_{0} \end{array}\right),$$

where *q*_0_ = λ^−2^(*γ*^2^*μ*_f_ − *pλ*^2^). To first order, we use the the equilibrium equation

4.20 div T=0,

to find

4.21 $$T^{\left(1\right)}=\left(\begin{array}{cc} -\frac{2k\mu \cos(kx)\lambda^{2}}{\gamma^{4}}\frac{dh_{f}^{\left(1\right)}}{dY}-q_{1}(Y) & -\frac{\mu \sin(kx)}{\gamma^{2}\lambda }\left(k^{2}h_{1}(Y)\lambda^{2}+\frac{d^{2}h_{f}^{\left(1\right)}}{dY^{2}}\right)\\ -\frac{\mu \sin(kx)}{\gamma^{2}\lambda }\left(k^{2}h_{1}(Y)\lambda^{2}+\frac{d^{2}h_{f}^{\left(1\right)}}{dY^{2}}\right) & \frac{2k\mu \cos(kx)}{\lambda^{2}}\frac{dh_{f}^{\left(1\right)}}{dY}-q_{1}(Y) \end{array}\right),$$

where

4.22 $$q_{1}=\frac{\mu_{f}}{\gamma^{4}k}\cos(kx)\left(\frac{d^{3}h_{f}^{\left(1\right)}}{dY^{3}}-k^{2}\lambda^{2}\frac{dh_{f}^{\left(1\right)}}{dY}\right).$$

Substituting these expressions into the first-order traction condition

4.23 $$T^{\left(0\right)}\cdot n^{\left(1\right)}+T^{\left(1\right)}\cdot n^{\left(0\right)}=pn^{\left(1\right)},$$

yields precisely (A 9a) and (A 9b). We conclude that the pressure has no effect on the linear analysis of the system: a bilayer develops the same wrinkling instability regardless of the pressure.

### (d) Fibre-reinforced substrate

A last modification we make to the bilayer system is to introduce embedded elastic fibres into the elastic substrate, as considered in [10]. This adds an orientational anisotropy into the system that mimics structures seen in many biological materials. For simplicity, we restrict our attention to the case of a single family of fibres with a vertical orientation and no pre-stretch. To describe the energetic cost of deforming the fibres, we add the following term to the energy density function [43,44]:

4.24 $$W_{r}(A)=m{\left((A\cdot n)\cdot (A\cdot n)-1\right)}^{2},$$

where **n** is a vertical unit vector in the reference configuration and *m* quantifies both the stiffness of the fibres and their volume fraction. As this modification changes the bulk energy, it has a corresponding effect on the Euler–Lagrange equation for the substrate. After perturbation and periodic decomposition, it reads:

4.25 $$\left(\lambda^{2}-4m(1-\lambda^{-2})\right)\frac{d^{4}h_{s}^{\left(1\right)}}{dY^{4}}-k^{2}\left(1+\lambda^{4}-4m(1-3\lambda^{-2})\right)\frac{d^{2}h_{s}^{\left(1\right)}}{dY^{2}}+k^{4}\lambda^{2}h_{s}^{\left(1\right)}=0.$$

Similarly, the traction boundary conditions at the film-substrate interface become

4.26*a* $$\begin{array}{rl} & \beta \left(\lambda \frac{d^{3}h_{f}^{\left(1\right)}}{dY^{3}}(0)-k^{2}\lambda^{-1}(2\gamma^{4}+\lambda^{4})\frac{dh_{f}^{\left(1\right)}}{dY}(0)\right)\\ & \;\;-\gamma^{4}\left((\lambda -4m(\lambda -\lambda^{-1}))\frac{d^{3}h_{s}^{\left(1\right)}}{dY^{3}}(0)-k^{2}\lambda^{-1}(2+\lambda^{4}-8m(1-2\lambda^{-2}))\frac{dh_{s}^{\left(1\right)}}{dY}(0)\right)=0, \end{array}$$

4.26*b* $$\begin{array}{rl} & \beta \left(\lambda \frac{d^{2}h_{f}^{\left(1\right)}}{dY^{2}}(0)+k^{2}\gamma^{4}\lambda^{-1}h_{f}^{\left(1\right)}(0)\right)-\gamma^{2}\left(1-4m(1-\lambda^{-2})\right)\left(\lambda \frac{d^{2}h_{s}^{\left(1\right)}}{dY^{2}}(0)+k^{2}\lambda^{-1}h_{s}^{\left(1\right)}(0)\right)=0. \end{array}$$

#### (i) Compression

In the compression case, the addition of fibres initially seems to have a limited effect. Compared to the unmodified case with the same large stiffness ratio, adding fibres with 0 < *m* < 1 causes the critical wavenumber to increase, the critical compression ratio to decrease and has no effect on the large *k* asymptote.

**Figure 15.**
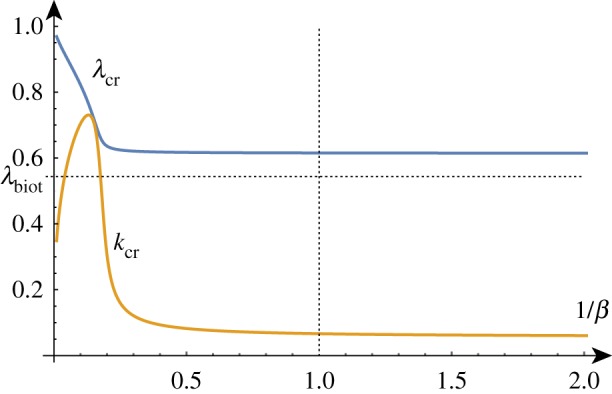
*k*_cr_ and λ_cr_ as functions of *β*^−1^ for *m* = 5/9. (Online version in colour.)

However, as we vary the stiffness ratio, we see some markedly different behaviour in the evolution of the critical point as a function of *β* (demonstrated in figure 15). For stiffness ratios *β* < *β*_c_ ≈ 8, the critical wavenumber rapidly decreases. This local maximum close to *k* = 0 persists even when *β* > 1 (at *β* = 1 we have no length scale and the wavelength is again undetermined). The position of the critical compression ratio does not appear to degenerate to λ_biot_ for small values of *β*.

**Figure 16.**
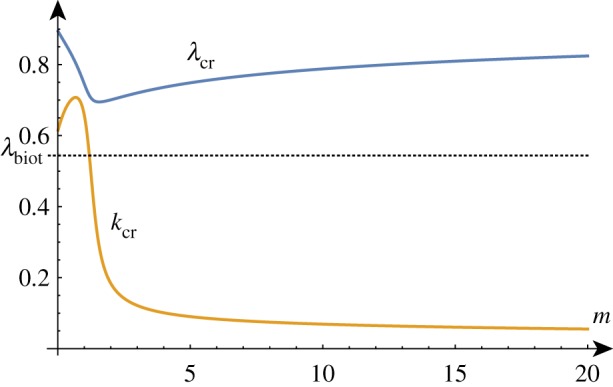
*k*_cr_ and λ_cr_ as functions of *m* for *β* = 10. (Online version in colour.)

Increasing the fibre stiffness parameter *m* (shown in figure 16) has a similar effect; for fibres stiffer than *m* = *m*_c_ ≈ 0.67, the critical wavenumber becomes close to *k* = 0.

To summarize, for fibres significantly stiffer than the elastic substrate in which they are embedded, we see a lower wavenumber wrinkling pattern emerge.

#### (ii) Growth

**Figure 17.**
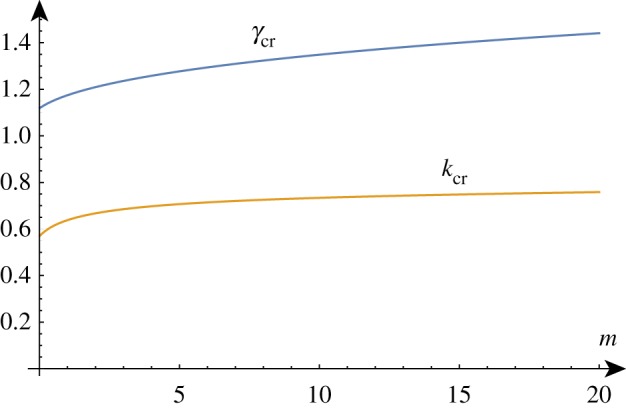
*k*_cr_ and *γ*_cr_ as functions of *m* for *β* = 10. (Online version in colour.)

In the growth case, the addition of fibres causes both the critical wavenumber and the critical growth factor to increase slightly but the large *k* behaviour of the system is unchanged. The critical growth factor and wavenumber have a similar qualitative behaviour compared to the unmodified case with the notable characteristic that the stiffness ratio 1/*β*_min_ at which the wavenumber blows up is significantly reduced. Plotting the dependence of the critical growth factor and wavenumber on the fibre stiffness parameter *m* as in figure 17 shows a gradual increase in both quantities as the fibres become stiffer. For a fixed *β*, there exists a finite (but extremely large) *m* such that *γ*_cr_ = *γ*_biot_ and *k*_cr_ becomes infinite.

## 5. Conclusion

We have presented a complete linear analysis for the plane-strain wrinkling of a film on an elastic substrate in the case of lateral compression and film growth. The analysis does not make any approximation on the thinness of the film or the relative stiffness ratio between substrate and film. Hence, it can be used as a general benchmark for approximate theories and identify their domain of validity. We also considered the role of secondary effects such as surface tension, pressure and fibres.

Our analysis further establishes that for films that are much stiffer than the substrate, a regular asymptotic expression in powers of 1/*β* leads to accurate predictions for the critical parameter and critical wavenumber selected at the wrinkling instability even when supplementary effects are considered. A rule of thumb is that for *β* ≳10, a 3-term expansion is sufficient in all cases to capture the correct behaviour. It also suggests that in this regime, approximate theories (beams and plates) may be sufficient as long as they correctly model the effect of the substrate. Our analysis can be used to gauge this calibration by matching the asymptotic behaviours of a plate or beam to the ones derived here.

As *β* decreases, a number of different effects appear that make general conclusions harder to reach. Depending on both the loading and the effect considered, qualitatively different behaviours are observed. For instance, the addition of any surface tension in compression changes the Biot surface instability (*k*_cr_ → ∞) to a Euler-type instability (*k*_cr_ → 0). Yet, the Biot instability is still the first selected for a growing film. Similarly, the minimal value *β*_min_ at which a linear instability is found depends greatly on both the loading and extra surface effects. It is, therefore, harder to obtain a general picture for the bifurcation of soft films on substrate. Yet, the linear analysis may not even be relevant in that regime for two reasons.

First, the film may undergo a creasing instability for values of the axial stretch around λ = 1/*γ* ≈ 0.64 [35]. Hence, the linear unstable wrinkling mode may not be observed past that critical value. Whether this instability is universally observed in bilayers and always selected is still an open problem.

Second, the analysis performed here is only linear and does not allow us to conclude about the existence of periodic solutions past the bifurcation point. The main problem is that the wrinkling instability may be supercritical or subcritical depending on the stiffness ratio [9,45–48]. Previous studies suggest that for sufficiently stiff films, the wrinkling instability is supercritical. The question is then to determine the value of *β* at which this supercritical bifurcation becomes subcritical and whether this value occurs before or after the Biot instability or the wrinkling instability. However, to answer this question and have a full picture of the wrinkling instability requires a full weakly nonlinear analysis of the bifurcation we have identified here. We leave this exercise for the second instalment of this work.

## Data accessibility

This article has no additional data.

## Appendix A. Appendix A: Boundary conditions

**(a) Regular case**

The boundary conditions for the stream functions are

A 1 $$\begin{array}{rl} & \lambda \frac{\partial^{3}\Psi_{f}^{\left(1\right)}}{\partial Y^{3}}(x,1)+\lambda^{-1}(2\gamma^{4}+\lambda )\frac{\partial^{3}\Psi_{f}^{\left(1\right)}}{\partial x^{2}\partial Y}(x,1)=0, \end{array}$$

A 2 $$\begin{array}{rl} & \lambda \frac{\partial^{2}\Psi_{f}^{\left(1\right)}}{\partial Y^{2}}(x,1)-\lambda^{-1}\gamma^{4}\frac{\partial^{2}\Psi_{f}^{\left(1\right)}}{\partial x^{2}}(x,1)=0, \end{array}$$

A 3 $$\begin{array}{rl} & \frac{\partial \Psi_{f}^{\left(1\right)}}{\partial x}(x,0)-\frac{\partial \Psi_{s}^{\left(1\right)}}{\partial x}(x,0)=0, \end{array}$$

A 4 $$\begin{array}{rl} & \frac{\partial \Psi_{f}^{\left(1\right)}}{\partial Y}(x,0)-\gamma^{2}\frac{\partial \Psi_{s}^{\left(1\right)}}{\partial Y}(x,0)=0, \end{array}$$

A 5 $$\begin{array}{rl} & \beta \left(\lambda \frac{\partial^{3}\Psi_{f}^{\left(1\right)}}{\partial Y^{3}}(x,0)+\lambda^{-1}(2\gamma^{4}+\lambda^{4})\frac{\partial^{3}\Psi_{f}^{\left(1\right)}}{\partial x^{2}\partial Y}(x,0)\right)\\ & \;\;-\gamma^{4}\left(\lambda \frac{\partial^{3}\Psi_{s}^{\left(1\right)}}{\partial Y^{3}}(x,0)+\lambda^{-1}(2+\lambda^{4})\frac{\partial^{3}\Psi_{s}^{\left(1\right)}}{\partial x^{2}\partial Y}(x,0)\right)=0, \end{array}$$

A 6 $$\begin{array}{rl} & \beta \left(\lambda \frac{\partial^{2}\Psi_{f}^{\left(1\right)}}{\partial Y^{2}}(x,0)-\gamma^{4}\lambda^{-1}\frac{\partial^{2}\Psi_{f}^{\left(1\right)}}{\partial x^{2}}(x,0)\right)\\ & \;\;-\gamma^{2}\left(\lambda \frac{\partial^{2}\Psi_{s}^{\left(1\right)}}{\partial Y^{2}}(x,0)-\lambda^{-1}\frac{\partial^{2}\Psi_{s}^{\left(1\right)}}{\partial x^{2}}(x,0)\right)=0, \end{array}$$

A 7 $$\begin{array}{rl} & \;\lim_{Y\to -\infty }\Psi_{s}^{\left(1\right)}(x,Y)=0, \end{array}$$

A 8 $$\begin{array}{rl} & \;\lim_{Y\to -\infty }\frac{\partial \Psi_{s}^{\left(1\right)}}{\partial Y}(x,Y)=0. \end{array}$$

After substitution of ***Ψ***^(1)^(*x*, *Y* ) = sin(*kx*)*h*^(1)^(*Y* ) for some *k* > 0, these boundary conditions read

A 9*a* $$\begin{array}{rl} & \lambda \frac{d^{3}h_{f}^{\left(1\right)}}{dY^{3}}(1)-k^{2}\lambda^{-1}(2\gamma^{4}+\lambda^{4})\frac{dh_{f}^{\left(1\right)}}{dY}(1)=0, \end{array}$$

A 9*b* $$\begin{array}{rl} & \lambda \frac{d^{2}h_{f}^{\left(1\right)}}{dY^{2}}(1)+k^{2}\gamma^{4}\lambda^{-1}h_{f}^{\left(1\right)}(1)=0, \end{array}$$

A 9*c* $$\begin{array}{rl} & h_{f}(0)-h_{s}(0)=0, \end{array}$$

A 9*d* $$\begin{array}{rl} & \frac{dh_{f}^{\left(1\right)}}{dY}(0)-\gamma^{2}\frac{dh_{s}^{\left(1\right)}}{dY}(0)=0, \end{array}$$

A 9*e* $$\begin{array}{rl} & \beta \left(\lambda \frac{d^{3}h_{f}^{\left(1\right)}}{dY^{3}}(0)-k^{2}\lambda^{-1}(2\gamma^{4}+\lambda^{4})\frac{dh_{f}^{\left(1\right)}}{dY}(0)\right)\\ & \;\;-\gamma^{4}\left(\lambda \frac{d^{3}h_{s}^{\left(1\right)}}{dY^{3}}(0)-k^{2}\lambda^{-1}(2+\lambda^{4})\frac{dh_{s}^{\left(1\right)}}{dY}(0)\right)=0, \end{array}$$

A 9*f* $$\begin{array}{rl} & \beta \left(\lambda \frac{d^{2}h_{f}^{\left(1\right)}}{dY^{2}}(0)+k^{2}\gamma^{4}\lambda^{-1}h_{f}^{\left(1\right)}(0)\right)-\gamma^{2}\left(\lambda \frac{d^{2}h_{s}^{\left(1\right)}}{dY^{2}}(0)+k^{2}\lambda^{-1}h_{s}^{\left(1\right)}(0)\right)=0, \end{array}$$

A 9*g* $$\begin{array}{rl} & \;\lim_{Y\to -\infty }h_{s}^{\left(1\right)}(Y)=0, \end{array}$$

A 9*h* $$\begin{array}{rl} & \;\lim_{Y\to -\infty }\frac{dh_{s}^{\left(1\right)}}{dY}(Y)=0. \end{array}$$

**(b) Boundary conditions for an upper substrate**

In the presence of an upper layer, the boundary conditions must be transformed as follows with boundary conditions given by

A 10*a* $$\begin{array}{rl} & h_{f}(1)-h_{t}(1)=0, \end{array}$$

A 10*b* $$\begin{array}{rl} & \frac{dh_{f}^{\left(1\right)}}{dY}(1)-\gamma^{2}\frac{dh_{t}^{\left(1\right)}}{dY}(1)=0, \end{array}$$

A 10*c* $$\begin{array}{rl} & \beta_{f}\left(\lambda^{2}\frac{d^{3}h_{f}^{\left(1\right)}}{dY^{3}}(1)-k^{2}(2\gamma^{4}+\lambda^{4})\frac{dh_{f}^{\left(1\right)}}{dY}(1)\right)-\beta_{t}\gamma^{4}\left(\lambda^{2}\frac{d^{3}h_{t}^{\left(1\right)}}{dY^{3}}(1)-k^{2}(2+\lambda^{4})\frac{dh_{t}^{\left(1\right)}}{dY}(1)\right)=0, \end{array}$$

A 10*d* $$\begin{array}{rl} & \beta_{f}\left(\lambda^{2}\frac{d^{2}h_{f}^{\left(1\right)}}{dY^{2}}(1)+k^{2}\gamma^{2}h_{f}^{\left(1\right)}(1)\right)-\beta_{t}\gamma^{2}\left(\lambda^{2}\frac{d^{2}h_{t}^{\left(1\right)}}{dY^{2}}(1)+k^{2}h_{t}^{\left(1\right)}(1)\right)=0, \end{array}$$

A 10*e* $$\begin{array}{rl} & \;\lim_{Y\to \infty }h_{t}^{\left(1\right)}(Y)=0, \end{array}$$

A 10*f* $$\begin{array}{rl} & \;\lim_{Y\to \infty }\frac{dh_{t}^{\left(1\right)}}{dY}(Y)=0, \end{array}$$

A 10*g* $$\begin{array}{rl} & h_{f}(0)-h_{s}(0)=0, \end{array}$$

A 10*h* $$\begin{array}{rl} & \frac{dh_{f}^{\left(1\right)}}{dY}(0)-\gamma^{2}\frac{dh_{s}^{\left(1\right)}}{dY}(0)=0, \end{array}$$

A 10*i* $$\begin{array}{rl} & \beta_{f}\left(\lambda^{2}\frac{d^{3}h_{f}^{\left(1\right)}}{dY^{3}}(0)-k^{2}(2\gamma^{4}+\lambda^{4})\frac{dh_{f}^{\left(1\right)}}{dY}(0)\right)-\gamma^{4}\left(\lambda^{2}\frac{d^{3}h_{s}^{\left(1\right)}}{dY^{3}}(0)-k^{2}(2+\lambda^{4})\frac{dh_{s}^{\left(1\right)}}{dY}(0)\right)=0, \end{array}$$

A 10*j* $$\begin{array}{rl} & \beta_{f}\left(\lambda^{2}\frac{d^{2}h_{f}^{\left(1\right)}}{dY^{2}}(0)+k^{2}\gamma^{2}h_{f}^{\left(1\right)}(0)\right)-\gamma^{2}\left(\lambda^{2}\frac{d^{2}h_{s}^{\left(1\right)}}{dY^{2}}(0)+k^{2}h_{s}^{\left(1\right)}(0)\right)=0, \end{array}$$

A 10*k* $$\begin{array}{rl} & \;\lim_{Y\to -\infty }h_{s}^{\left(1\right)}(Y)=0, \end{array}$$

A 10*l* $$\begin{array}{rl} & \;\lim_{Y\to -\infty }\frac{dh_{s}^{\left(1\right)}}{dY}(Y)=0. \end{array}$$
